# Natural variation in stomatal response to closing stimuli among *Arabidopsis thaliana* accessions after exposure to low VPD as a tool to recognize the mechanism of disturbed stomatal functioning

**DOI:** 10.1093/jxb/eru370

**Published:** 2014-09-09

**Authors:** Sasan Aliniaeifard, Uulke van Meeteren

**Affiliations:** ^1^Horticultural Production Chains, Department of Plant Sciences, Wageningen University, p.o. Box 630, 6700 AP Wageningen, The Netherlands; ^2^Department of Horticulture, College of Abureyhan, University of Tehran, PC. 3391653775, Pakdasht, Tehran, Iran

**Keywords:** *Arabidopsis thaliana*, stomata, vapour pressure deficit (VPD), abscisic acid, natural variation, desiccation.

## Abstract

Stomatal responses to closing stimuli are disturbed after long-term exposure of plants to low vapour pressure deficit (VPD). The mechanism behind this disturbance is not fully understood. Genetic variation between naturally occurring ecotypes can be helpful to elucidate the mechanism controlling stomatal movements in different environments. We characterized the stomatal responses of 41 natural accessions of *Arabidopsis thaliana* to closing stimuli (ABA and desiccation) after they had been exposed for 4 days to moderate VPD (1.17 kPa) or low VPD (0.23 kPa). A fast screening system was used to test stomatal response to ABA using chlorophyll fluorescence imaging under low O_2_ concentrations of leaf discs floating on ABA solutions. In all accessions stomatal conductance (g_s_) was increased after prior exposure to low VPD. After exposure to low VPD, stomata of 39 out of 41 of the accessions showed a diminished ABA closing response; only stomata of low VPD-exposed Map-42 and C24 were as responsive to ABA as moderate VPD-exposed plants. In response to desiccation, most of the accessions showed a normal stomata closing response following low VPD exposure. Only low VPD-exposed Cvi-0 and Rrs-7 showed significantly less stomatal closure compared with moderate VPD-exposed plants. Using principle component analysis (PCA), accessions could be categorized to very sensitive, moderately sensitive, and less sensitive to closing stimuli. In conclusion, we present evidence for different stomatal responses to closing stimuli after long-term exposure to low VPD across *Arabidopsis* accessions. The variation can be a useful tool for finding the mechanism of stomatal malfunctioning.

## Summary

There is a remarkable natural variation in stomatal response to closing stimuli in *Arabidopsis*, which can be exploited for studying the mechanism of stomatal malfunctioning after exposure to low VPD.

## Introduction

Stomata pores in the epidermis of leaves are largely responsible for gas exchange, especially CO_2_ uptake and water loss, between plant and atmosphere. A fine regulation of the stomata aperture is required to allow sufficient CO_2_ uptake for photosynthesis, while preventing excessive water loss through transpiration under various environmental conditions.

It is well known that as a short-term response, stomata widen their aperture when the atmospheric vapour pressure deficit (VPD) is low and decrease the aperture after an increase of VPD (Outlaw and De Vlieghere-He, 2001; Shope *et al.*, 2008; Okamoto *et al.*, 2009; Aliniaeifard and van Meeteren, 2013; Aliniaeifard *et al.*, 2014). The mechanism of stomatal responses to VPD has been the subject of many studies during the last few decades (Farquhar, 1978; Appleby and Davies, 1983; Assmann and Gershenson, 1991; Mott and Parkhorst, 1991; Bunce, 1997; Mott and Peak, 2012; Fanourakis *et al.*, 2013). ‘Feedforward’ and ‘feedback’ hypotheses have been proposed for the stomatal response to VPD (Farquhar, 1978; Grantz, 1990; Saliendra *et al.*, 1995). In the ‘feedforward’ hypothesis, the stomatal response to VPD is a result of direct sensing of the VPD, and is independent from leaf water status (Farquhar, 1978; Franks *et al.*, 1997). It was shown that ABA can act as intermediary between stomatal responses and VPD (Grantz, 1990; Zhang and Davies, 1991; Bunce, 1998; Tardieu and Simonneau, 1998). A close relationship has been observed between VPD and the ABA level in the leaf. Increasing VPD results in ABA accumulation in the leaf (Bauerle *et al.*, 2004) and decreasing VPD causes catabolism of ABA (Okamoto *et al.*, 2009). Mott and Parkhurst (1991) proposed that stomata respond to VPD via transpiration rate rather than humidity *per se*. In the ‘feedback’ hypothesis, stomatal response to VPD is a result of a negative feedback of transpiration on leaf water status (Raschke, 1970; Saliendra *et al.*, 1995). In this hypothesis, indirect induction of ABA production by increased transpiration has been proposed (Buckley, 2005). However, the involvement of ABA in the stomatal response to VPD is still debated. Assmann *et al.* (2000) showed both ABA insensitive (*abi1-1* and *abi2-1*) and ABA-deficient mutants (*aba1*) of *Arabidopsis* have a similar stomatal response to increased VPD compared with wild-type *Arabidopsis* plants, which make the role of ABA more complicated. Recently feedback and feedforward mechanisms together have been taken into account for stomatal response to VPD (Peak and Mott, 2011). Accordingly, a dual role for ABA-induced stomatal closure has been proposed: (i) a direct biochemical mechanism on guard cells of stomata and (ii) an indirect effect of ABA through a decreased leaf hydraulic conductance (Pantin *et al.*, 2013).

In all the mentioned studies, the short-term response of the stomata to VPD was investigated, and the focus was on the stomatal response to high VPD. However, when plants were grown at low VPD, the behaviour of the stomata in response to desiccation or ABA changed and the stomata showed a diminished response to closing stimuli (Fordham *et al*., 2001a, b; Rezaei Nejad and van Meeteren, 2005, 2007, 2008; Rezaei Nejad *et al.*, 2006; Fanourakis *et al.*, 2011; Arve *et al.*, 2012; Aliniaeifard and van Meeteren, 2013; Aliniaeifard *et al.*, 2014). Even when full-grown leaves were transferred from high to low VPD this loss of stomatal response to closing stimuli could be induced (Rezaei Nejad and van Meeteren, 2008). The occurrence of stomatal malfunctioning depends on the duration of the exposure to low VPD and it is species dependent (Fanourakis *et al.*, 2011; Aliniaeifard and van Meeteren, 2013; Aliniaeifard *et al.*, 2014). We previously proposed that after prolonged exposure to low VPD a perturbation in the ABA signalling pathway inside the guard cells leads to the malfunctioning of the stomata. However, the altered signalling pathway in the guard cells of dysfunctional stomata is still unknown (Aliniaeifard and van Meeteren, 2013).

Variation in sensitivity of stomatal conductance to VPD has been observed at intraspecific levels. In red maple, for example, wet site ecotypes responded quicker to water stress than dry site ecotypes by biosynthesizing ABA and by closing their stomata (Bauerle *et al.*, 2004). *Arabidopsis* is widely distributed around the world and large variation has been found in this species for many aspects. Genetic variation between accessions of *Arabidopsis* under stress conditions has been found for responses to high light (Jung and Niyogi, 2009; Athanasiou *et al.*, 2010), ozone (Brosché *et al.*, 2010), freezing (Hannah *et al.*, 2006), drought (Bouchabke *et al.*, 2008), high temperature (Edwards *et al.*, 2006), and salinity (Katori *et al.*, 2010). Brosché *et al*. (2010) investigated the ozone sensitivity between *Arabidopsis* accessions and correlated it to stomatal conductance. Bouchabke *et al.* (2008) showed differences in cut rosette water loss between accessions under drought stress and assumed that these differences were related to differences in stomatal aperture. The ABA signalling pathway in guard cells comprises a network of many components. To find the effect of prior exposure to low VPD on guard cell signalling, it will be very helpful to identify variation in stomatal response to closing stimuli in a collection of *Arabidopsis* accessions after exposure of the plants to different VPDs. However, to the best of our knowledge there is not any publication available in relation to natural variation in the stomatal response of *Arabidopsis* to closing stimuli nor in the stomatal response after prolonged exposure to different VPDs.

In this paper, we analysed the stomatal response of 41 distinct accessions of *Arabidopsis* to ABA and to desiccation after growing them at moderate VPD as well as after transfer of the plants for 4 d to low VPD. For efficient large-scale screening of stomatal responses to ABA in plants that have been exposed to different environmental conditions, we developed a system in which we used chlorophyll fluorescence imaging under a non-photorespiratory condition for leaf discs floating on ABA solutions. For screening of stomatal responses to desiccation we used the rate of water loss as function of leaf relative water content (RWC) from excised leaves to characterize the water loss parameters of the *Arabidopsis* accessions. We asked the following questions: (i) how large is the variation in the stomatal response of the *Arabidopsis* accessions to closing stimuli (ABA and desiccation) after acclimation to moderate (M) and low (L) VPDs? (ii) Are there relationships between the stomatal conductance after long-term exposure to M and L conditions (without closing stimuli) and responses of the stomata to closing stimuli? (iii) Is there a relationship between foliar ABA content before or during desiccation after long-term exposure to M and L conditions and stomatal response to closing stimuli?

## Materials and methods

**Fig. 1.**
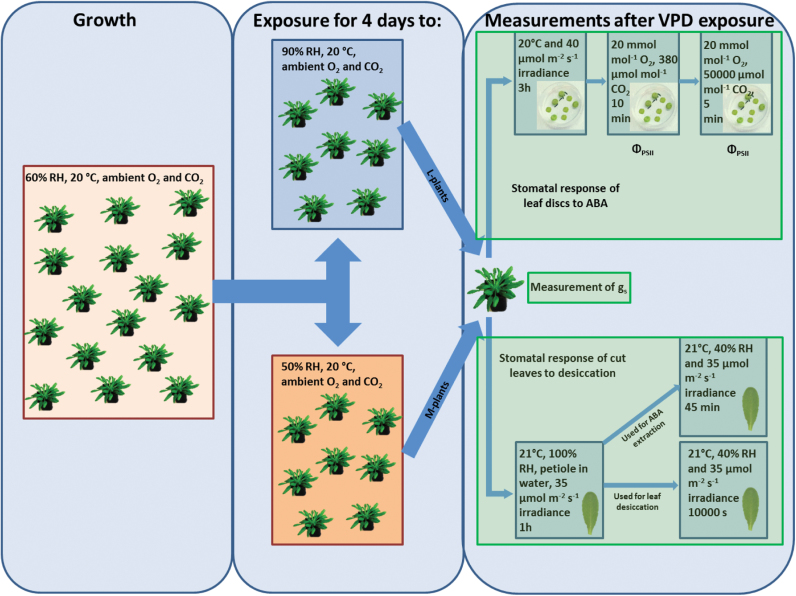
Schematic representation of the experimental setup and conditions which were used for growing plants and measurements. Boxes describe the conditions used for growing plants and measurements. The arrows shows transferring to new conditions.

An overview of treatments and measurements is given in Fig. 1

### Plant material and growth conditions

Forty-one natural accessions of *Arabidopsis thaliana* obtained from the *Arabidopsis* Biological Resource Center (ABRC), Ohio State University, USA were used. The accessions were chosen in such a way that there was a large genetic and geographical diversity among them. The names and geographical characterizations of the accessions are given in Table 1.

**Table 1.** Geographical characterizations of the accessions used in the current experiment

Accession	PCA number	ABRC Stock number	Latitude	Longitude	Altitude (m)	Temperature (°C)	Precipitation (mm)	Country
1-pn	1	CS76197	50	10	289	9.1	45.6	Germany
Aa-0	2	CS28007	50.9	9.5	297	8.6	58	Germany
Ag-0	3	CS76087	45	1.3	299	13.3	60.6	France
Bur-0	4	CS76105	54.1	–6.2	8	9.1	72.4	Ireland
C24	5	CS76106	41.2	–8.4	227	14.8	96.6	Portugal
Bs-2	6	CS28097	47.5	7.5	331	10.9	62.5	Switzerland
Cvi-0	7	CS76116	15.1	–23.6	304	24.7	17.7	Cape Verde island
Eri-1	8	CS22548	56.4	15.3	118	7.5	46.3	Sweden
Ler-1	9	CS76164	52.7	15.2	19	9.3	45.1	Poland
Lis-1	10	CS76169	56	14.7	0	8.5	31.2	Sweden
Lis-2	11	CS76170	56	14.7	0	8.5	31.2	Sweden
Lm-2	12	CS76173	48	0.5	146	12.3	54.5	France
Lp2-2	13	CS76176	49.3	16.8	567	9.5	41.2	Czech republic
Map-42	14	CS76180	42.1	–86.4	194	9.2	78.5	USA
Mib-15	15	CS76181	47.3	5.3	208	11.3	60.8	France
MNF-Pot-68	16	CS76188	43.5	–86.2	243	11.4	53.1	USA
Mt-0	17	CS76192	32.3	22.4	283	18.2	16.5	Libya
Mz-0	18	CS76193	50.3	8.3	343	11.1	32.8	Germany
NFA-10	19	CS76198	51.4	–0.6	63	9.9	55.2	UK
Ost-0	20	CS76202	60.2	18.3	24	6.2	37.8	Sweden
Pa-1	21	CS76204	38	13.2	938	18.9	65.5	Italy
Par-5	22	CS76207	46.6	–0.2	206	11.9	52.6	France
Pent-1	23	CS76209	43.7	–86.3	187	9.3	67.5	USA
Per-1	24	CS76210	58	56.3	135	2.5	28.75	Russia
Petergof	25	CS76211	59	29	74	5.9	63.6	Russia
Pla-0	26	CS28640	41.5	2.2	222	16.4	50.2	Spain
Pog-0	27	CS28650	49.2	–123.2	71	10.7	101.3	Canada
Pro-0	28	CS76214	43.2	–6	324	13.3	79.8	Spain
Pu2-23	29	CS76215	49.4	16.3	492	9.4	41.1	Czech republic
Ren-1	30	CS76218	48.5	–1.4	40	12.1	46.7	France
Sapporo-0	31	CS28724	43	141.3	27	15.6	37.1	Japan
Shahdara	32	CS76227	38.3	68.4	646	15.7	37.3	Tajikistan
T10-60	33	CS76234	55.6	13.2	14	8	30.5	Sweden
Ta-0	34	CS76242	49.5	14.5	620	8.1	44.3	Czech republic
Ws-0	35	CS76303	52.3	30	132	7.5	55.1	Russia
Zdrl 2**–**25	36	CS76308	49.3	16.2	501	7.6	52.3	Czech republic
Col-0	37	CS76113	–	–	–	–	–	Unknown
Kas-1	38	CS76150	35	77	5301	2.3	13.1	India
Bay-0	39	CS76094	49	11	533	8.5	39.5	Germany
Ba-1	40	CS28053	56.5	–4.7	184	9	125	UK
RRS-7	41	CS28713	41.5	–86.4	220	8.4	76.7	USA

After stratification of seeds at 4 °C for 4 d, the seeds were sown in a pot filled with a soil developed for *Arabidopsis* (*Arabidopsis* soil, Horticoop, the Netherlands). After germination, in the stage of cotyledonous leaves, the plants were transplanted to pots (l×w×h= 7 cm×7 cm×7cm) (one plant per pot) filled with a mixture of fine and course sands. The bottom of the pots were covered with net-like plastic sheets and the top of the sand mixture was covered with 0.5cm *Arabidopsis* soil. The surface of the soil was covered with a black plastic sheet to prevent contact of the leaves with wet soil and to prevent evaporation from the soil surface which otherwise will cause a micro-climate with low VPD around the rosette of the plants. The plants were placed in a tray and irrigated four times per week using a nutrient solution developed for *Arabidopsis* (Van Iperen International, Westmaas, the Netherlands) (Supplementary Table S1). All plants were grown in a climate chamber with a constant temperature of 20±1 °C; 60±5% relative humidity (RH), resulting in a VPD of 0.94 kPa, 12h/12h day night lighting period; 150 µmol m^–2^ s^–1^ light (measured with an LI-250 light meter, Li-Cor, Lincoln, NE, USA) produced by fluorescent tubes (TLD 58W/84 Philips); and 380±20 µmol mol^–1^ CO_2_ (determined using Indoor Air Quality Meter, Model 8760, TSI Incorporated, Shoreview, USA). When the plants had produced fully developed leaves in the stage between 3.9 and 5 (stages as indicated by Boyes *et al.*, 2001), they were transferred to two other growth chambers (l×w×h=1.3 m×0.8 m×1 m; Weiss Technik, Germany) with the same temperature and light conditions but with different VPDs. One of them with 50±5% RH, resulting in a VPD of 1.17 kPa (M); another one with 90±5% RH, resulting in a VPD of 0.23 kPa (L). For each accession there were eight plants per growth chamber (VPD). Temperature and RH in the climate room and growth chambers were recorded every 5min using data loggers (Fourier MicroLog EC650, MicroDAQ.com, Ltd. Contoocook, New Hampshire, USA). After 4 d exposure to the two VPD conditions, fully developed leaves were used for analysing the response of stomata to ABA and desiccation.

### Stomatal conductance

Stomatal conductance (g_s_) was recorded in fully developed leaves of eight plants (one leave per plant) after a 4-day exposure to each VPD, using a porometer (Delta-T Devices Ltd, Cambridge, UK) in an environment with a 20 °C temperature, 50% RH and 150 µmol m^–2^ s^–1^ illumination.

### Mapping of stomatal response to ABA using chlorophyll fluorescence

To investigate the stomatal response of M- and L-exposed plants to ABA, chlorophyll fluorescence imaging under a non-photorespiratory condition (low O_2_ concentration) was used. Because PSII photochemical efficiency (Φ_PSII_) was measured while photorespiration was inhibited, a decreased Φ_PSII_ is closely related to stomatal closure (Rezaei Nejad *et al.*, 2006). However, this relationship is not always linear. To ensure that the decreased Φ_PSII_ was indeed due to stomatal closure, at the end of the imaging of Φ_PSII_ for the different treatments, Φ_PSII_ was measured in an atmosphere with high CO_2_ concentration (20 mmol mol^–1^ O_2_, 50 000 µmol mol^–1^ CO_2_) to test the recovery of Φ_PSII_. When stomatal closure occurs, it results in scarcity in CO_2_ in the stomatal cavity and as a result in low Φ_PSII_. In this situation, when decreased Φ_PSII_ is due to lack of internal CO_2_, a very high concentration of CO_2_ will be able to diffuse into the stomatal cavity (even when stomata are closed) and to restore the Φ_PSII_.

Leaf discs (0.5cm diameter) were prepared from eight leaves (one disc/leaf) of eight individual plants (one leaf/plant). The middle of the leaf between main vein and leaf margin was chosen for making the leaf discs. The leaf discs were placed with their abaxial surface up in petri dishes filled with stomata-opening medium (50mM KCl, 10mM MES-KOH, pH 6.15, 50 µM CaCl_2_ in degassed distilled water) with different concentrations of ABA (0, 50, 100, 200 µM ABA). To obtain fast and uniform uptake of the solutions, 3min vacuum infiltration (75 mbar) was used. After vacuum infiltration, the leaf discs were pre-incubated for 3h in the above mentioned ABA-solutions at 20 °C and 40 µmol m^–2^ s^–1^ irradiance. Thereafter, the petri dishes were placed in a flow-through cuvette. Four petri dishes could be placed simultaneously in the cuvette. The cuvette was placed under a chlorophyll fluorescence imaging system (FluorCam 700MF, PSI, Brno, Czech republic). The temperature in the cuvette was 22±1 °C. The imaging measurement was conducted while flowing an atmosphere with 20 mmol mol^–1^ O_2_, 380 µmol mol^–1^ CO_2_ and the rest N_2_ (non-photorespiratory condition) into the cuvette. The RH was set to 40±3% via passing the air in a temperature-controlled column of iron (II)-sulphate heptahyrate (Fluka). The leaf discs in the stomata-opening medium were exposed to a continuous irradiance of 100 µmol m^–2^ s^–1^. Preliminary experiments showed that 10min was sufficient to reach the steady state Φ_PSII_. Therefore, after 10min the protocol for the FluorCam was run and the average value of Φ_PSII_ per leaf disc was calculated by using version 5 of FluorCam software. Values for F_t_ and F_m_’ in the generated image were averaged over all pixels per leaf disc and the Φ_PSII_ was calculated using the ratio (F_m_’–F_t_)/F_m_’. To be sure that the VPD or other environmental conditions in the growth chambers had no negative effects on the plants, F_v_/F_m_ was measured of dark adapted plants of five accessions (Col-0, Cvi-0, Map-42, C24, and Rrs-7). The value of F_v_/F_m_ was around 0.8, confirming there is no stress due to growing the plants in the climate chambers used.

### Stomatal response to desiccation

To study the effect of desiccation on leaf transpiration rate of the *Arabidopsis* accessions, fully developed leaves from eight plants (one leaf/plant) were detached at the same time as sampling for Φ_PSII_ response to ABA and an image was taken to determine the leaf surface area. Then the leaves were placed in closed petri dishes with a layer of degassed deionized water. The leaves were incubated for 1h at 21 °C and 35 µmol m^–2^ s^–1^ irradiance (Fanourakis *et al.*, 2011). Under this condition the leaves gained maximum fresh weight. For desiccation, the leaves were removed from the petri dishes and placed with the abaxial side up on balances in a test room (40±3% RH, 21 °C, resulting in 1.40 kPa VPD and 35 µmol m^–2^ s^–1^ irradiance). The water loss of the leaves was recorded gravimetrically every 10 s for a period of 10 000 s. The leaf area was calculated by using the public domain image processing program ImageJ (ImageJ, U. S. National Institutes of Health, Bethesda, Maryland, USA, http://imagej.nih.gov/ij/). The transpiration rate was calculated according to equation 1.

Equation 1:

$$\begin{array}{l} \begin{array}{c} Transpiration\;rate\\ \left(mmol\;m^{-2}s^{-1}\right) \end{array}=(\left(\left(\frac{\Delta \;fresh\;weight\;\left(g\right)}{molar\;mass\;water\;\left(\frac{g}{mol}\right)}\right)\times 1000\left(\frac{mmol}{mol}\right)\right)\;\\ /measurement\;frequency\;(s))/\;leaf\;area\;(m^{2}) \end{array}$$

After the desiccation period, the leaves were dried for 48h at 80 °C. The relative water content (RWC) during the desiccation period was calculated according to Slavik (1974). As differences in the rate of water loss from cut leaves of different treatments will result in differences in leaf water content over time, changes in transpiration rate (E) were investigated in relation to RWC.

### ABA extraction and quantification

One fully developed leaf was detached from M- and L-exposed plants and incubated in a petri dish for 1h at 21 °C, 100% RH (VPD≈0). Three plants per treatment were used as repetitions. Samples for ABA analysis were taken before and after 45min desiccation of the leaves. For desiccation, the leaves were removed from petri dishes and then placed upside down in a test room (40±3% RH, 20 °C, resulting in 1.40 kPa VPD, and 35 µmol m^–2^ s^–1^ irradiance). 0.5g of leaf tissue was ground in a mortar using liquid nitrogen. The samples were extracted with 1ml of cold ethyl acetate containing [^2^H_6_]-ABA as internal standard to have 0.1 nmol internal standard in the extraction. The samples were vortexed (1min), then sonicated (15min) in a Branson 3510 ultrasonic bath (Branson Ultrasonics, Danbury, CT, USA). Samples were centrifuged for 10min at 2200rpm in an MSE Mistral 2000 centrifuge (Mistral Instruments, Leicester, UK). The supernatant was transferred to a 4-ml glass vial. The pellets were re-extracted with 1ml of methanol without sonication. The solvent fractions were pooled in a 4-ml glass vial. Then the samples were dried using a speedvac (SPD2010-230, Thermo Scientific, USA) and the residue was dissolved by 50 µl methanol. MQ water (3ml) was added to the samples and the extracts were purified using 500mg C18 columns. The samples were eluted with 1ml acetone. Then the acetone was evaporated under N_2_. The residue was dissolved in 200 µl of acetonitrile:water:formic acid (10:90:0.1, v:v:v). Samples were ﬁltered into vials with Minisart 0.2 µm ﬁlters (Sartorius, Goettingen, Germany) and were used for LC-MS ⁄MS analysis according to López‐Ráez *et al*. (2010).

### Statistical analysis

Data for stomatal response to ABA, ABA content, and g_s_ were subjected to analysis of variance (ANOVA) using factorial analysis. Treatment means were compared using least significant difference (LSD) test and *P*>0.05 was assumed as not significant. The change of transpiration rate (E) as a function of RWC was fitted using a sigmoidal dose-response curve with a variable slope [E=Bottom+((Top-Bottom)/(1+10^(RWC50–RWC).Slope^))]. The parameters RWC50 and the slope of the fitted curves were used for the analyses of ecotype differences in the relationship between transpiration rate and RWC. GraphPad Prism 5 for Windows (GraphPad software, Inc. San Diego, CA) and IBM SPSS Statistics version 19 were used for statistical analysis of the data. RWC50, slope, and stomatal response to 200 µmol ABA (as measured by changes in Φ_PSII_) for moderate and low VPD-exposed plants were used for principle component analysis (PCA) to compare the differences between accessions. The free software environment for statistical computing R (version 3.0.0) was used for PCA and hierarchical cluster classification.

## Results

### Stomatal conductance increased in all *Arabidopsis* accessions after prolonged exposure to low VPD

**Fig. 2.**
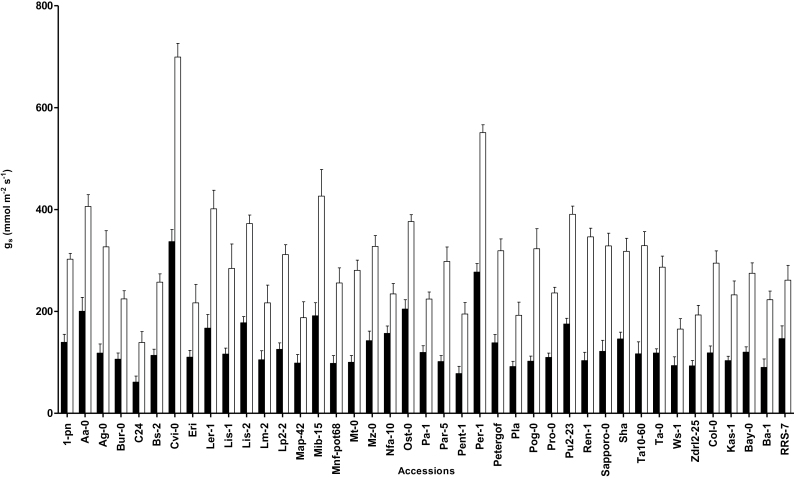
Stomatal conductance (g_s_) of 41 *Arabidopsis* accession after exposure to different VPDs. Plants had been exposed to moderate VPD (1.17 kPa; filled bars) or to low (0.23 kPa; open bars) VPD. The measurements were carried out at 1.40 kPa VPD and 35 µmol m^–2^ s^–1^ irradiance. g_s_ was recorded in fully developed leaves of eight plants (one leave per plant) after a 4-day exposure to each VPD. Bars represent the mean of eight leaves±standard error of the mean.

Prior exposure to low VPD (L) for 4 d caused a significant increase in stomatal conductance (g_s_) in all tested *Arabidopsis* accessions (Fig. 2). The relative effect of low VPD on g_s_ differed per accession (*P*=0.0001 for interaction between accession×VPD). Highest g_s_ among the studied accessions was found in Cvi-0 after exposure to L. Similarly, Cvi-0 showed highest g_s_ among *Arabidopsis* accessions that were not exposed to low VPD (M). The lowest g_s_ was observed in C24 in both M and L plants (Fig. 2).

### Stomata of 39 out of 41 *Arabidopsis* accessions strongly lost their responsiveness to ABA after prior exposure to low VPD

**Fig. 3.**
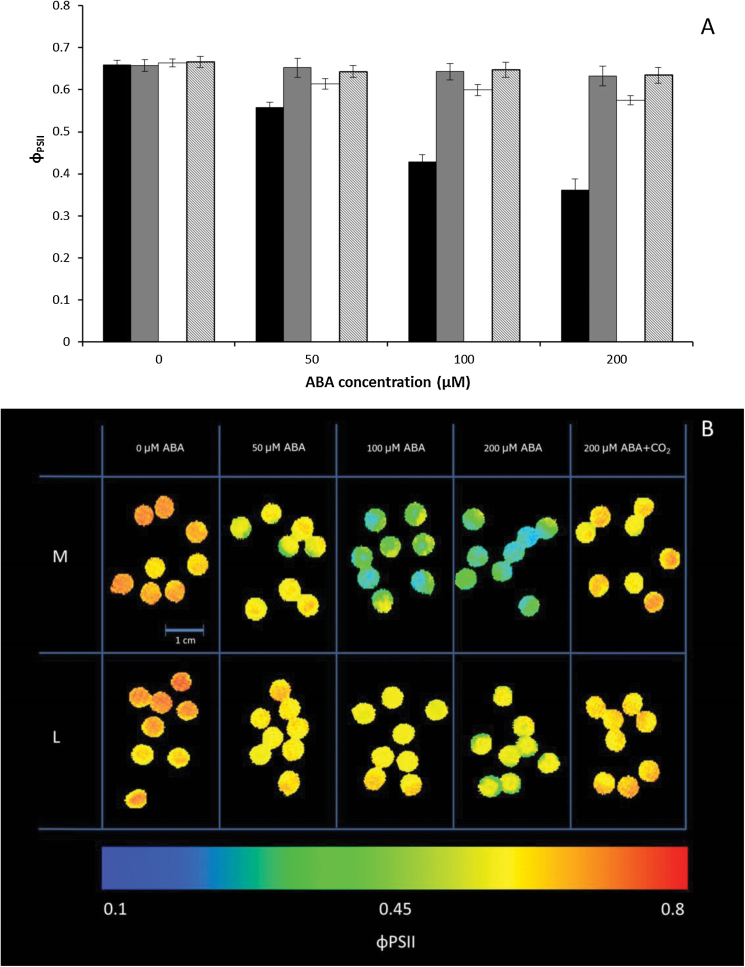
Average PSII efficiency (Φ_PSII_) (A) and representative images of Φ_PSII_ (B) for Col-0 leaf discs in response to ABA after prior exposure to different VPDs. Φ_PSII_ was measured under non-photorespiratory conditions (20 mmol mol^–1^ O_2_, 380 µmol mol^–1^ CO_2_ and remainder N_2_) in plants that had been exposed for 4 d to moderate (1.17 kPa; black bars in (A)) or to low (0.23 kPa; L; white bars in (A)) VPD in response to ABA. At the end, an image was made after 5min exposure to 20 mmol mol^–1^ O_2_ and 50000 µmol mol^–1^ CO_2_ (grey bars for M, cross-hatched bars for L in Fig. 3A and +CO_2_ in Fig. 3B). Leaf discs (0.5cm diameter) were put with the abaxial surface up in petri dishes filled with stomata-opening medium with different concentrations of ABA (0, 50, 100, 200 µM ABA), and Φ_PSII_ was recorded 3h after application of the ABA. Bars represent the mean of Φ_PSII_ of eight leaf discs±standard error of the mean.

**Fig. 4.**
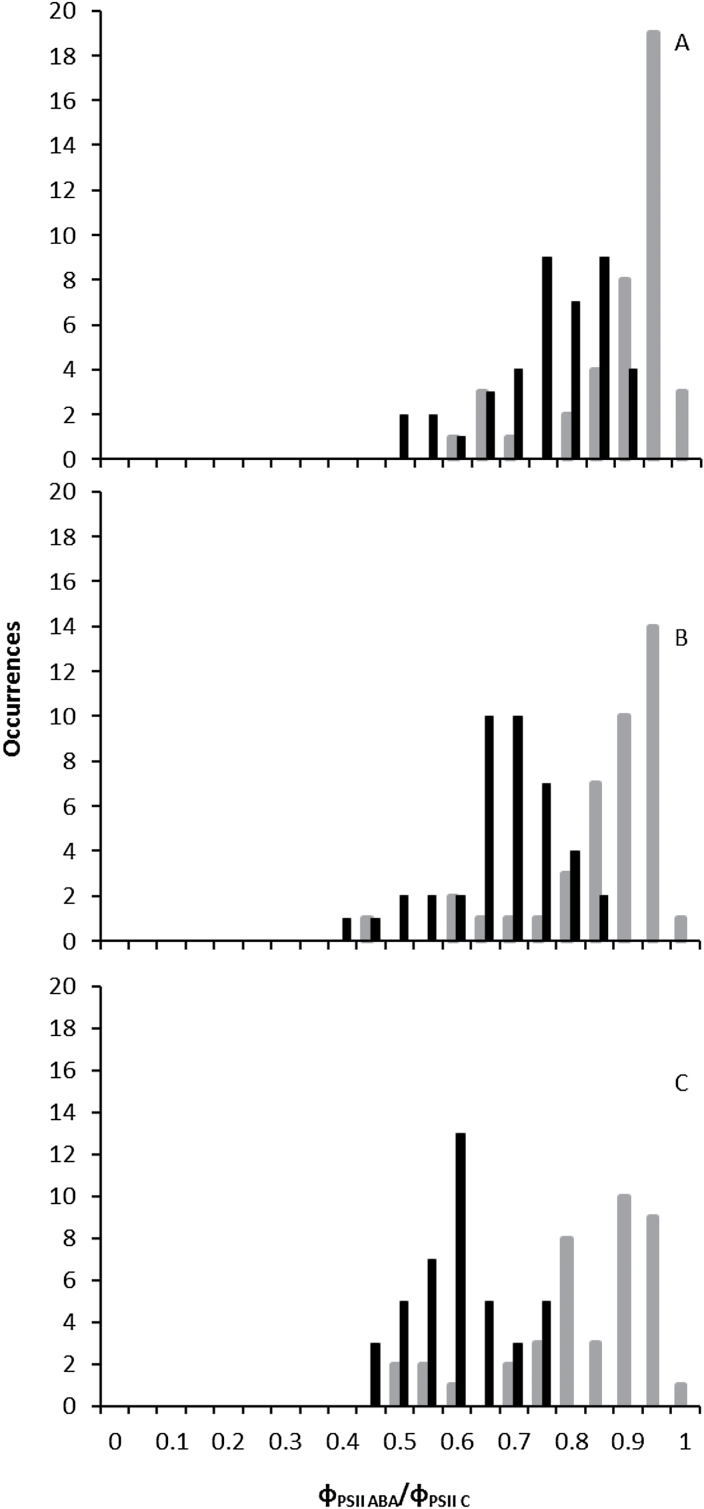
Frequency distribution of different accessions according to the relationships between PSII efficiency (Φ_PSII_) under non-photorespiratory conditions in response to 50 (A), 100 (B), and 200 µM ABA (C) relative to no ABA (Φ_PSII ABA_/ Φ_PSII C_) after 4 d exposure of plants to moderate VPD (1.17 kPa; black bars) or to low VPD (0.23 kPa; grey bars).

For most of the accessions, stomatal closure response to ABA was less after prior exposure to low VPD, as shown by higher PSII efficiency (Φ_PSII_) after ABA application compared with that of previously M-exposed plants, when measured under a non-photorespiratory condition. An example is given for Col-0 in Fig. 3A, B. Although the response to ABA was strongly affected by the previous VPD to which the plants had been exposed, the lowest Φ_PSII_ for both the L- and the M-exposed Col-0 was observed in 200 µM ABA. For all treatments, application of high CO_2_ (50 000 µmol mol^–1^ CO_2_) to the leaf discs resulted in the recovery of Φ_PSII_; this indicates that the reduction of Φ_PSII_ was mainly due to stomatal closure. The effect of different concentrations of ABA (50, 100, 200 µM) on Φ_PSII_ of the 41 *Arabidopsis* accessions that had been exposed for 4 d to M or L conditions can be seen in Supplementary Table S2 (*P*=0.001 for interaction between accession×200 µM ABA). To be able to compare the accessions, the effect of ABA was expressed in relative values as Φ_PSII x ABA_/Φ_PSII C_, which is the ratio of Φ_PSII_ measured of leaf discs at one of the ABA concentrations and Φ_PSII_ measured without ABA application. The ‘x’ indicates the ABA concentration in µM. Substantial variation was found in stomatal response to ABA among accessions after exposure to different VPDs (Fig. 4). By application of different ABA concentrations (Fig. 4A–C), heterogeneity was observed in Φ_PSII x ABA_/Φ_PSII C_ in both M and L plants. In 50 µM ABA, Φ_PSII 50 ABA_/Φ_PSII C_ in L-plants was partly overlapped by M plants (Fig. 4A). The overlapping accessions for their Φ_PSII ABA_/Φ_PSII C_ responses were decreased by increasing the ABA concentration to 100 (Fig. 4B) and 200 (Fig. 4C) µM ABA, and two distinct patterns of distribution between M and L plants were recognized. Especially at 200 µM ABA, the distribution for L plants was much broader than the distribution for M plants.

In all accessions the Φ_PSII ABA_/Φ_PSII C_ was decreased in an ABA concentration-dependent manner for both VPDs (Supplementary Table S2). Significant differences were found between M and L plants for Φ_PSII_ in response to ABA for 39 of the tested accessions. In all 39 accessions, the Φ_PSII_ was reduced less by ABA for L plants in comparison with what it was for M plants (Supplementary Table S2).

In contrast to the other accessions, in Map-42, C24, Pent1, Lis1, and Ost-0, the Φ_PSII_ of L plants strongly responded to ABA; that was also true for the lowest ABA concentration tested (50 µM). However, in Pent1, Lis1, and Ost-0, M and L plants significantly differed in their response to ABA. In the case of two accessions, Map-42 and C24, no significant differences were found between M and L plants for their response to ABA; for both plant types (M and L) the Φ_PSII_ showed a comparable strong decrease by application of ABA (Supplementary Table S2).

### Stomata of 39 out of 41 *Arabidopsis* accessions kept their responsiveness to desiccation after prior exposure to low VPD

Although during desiccation, the rate of water loss decreased in leaves of both M- and L-exposed accessions, in some of them the amount of water loss was higher in L-exposed compared with M-exposed plants. The transpiration rate (E) for M and L Col-0 and Cvi-0 is presented as an example of the water loss in response to desiccation (Supplementary Fig. S1). E followed an exponential decay over desiccation time. In both Col-0 and Cvi-0 significant differences were found during desiccation between plants exposed to M and L conditions. However, a larger difference was found between M and L exposed Cvi-0 during desiccation time in comparison with Col-0.

The influence of water status of the leaf during desiccation on the stomata opening was expressed using the relationship between E and RWC (E×RWC). In all accessions, E followed a sigmoidal decay as a function of RWC. RWC50 and the slope of the fitted curves of E×RWC were used to analyse the response of stomata to RWC during desiccation of the ecotypes after prior exposure to M and L conditions. Higher RWC50 or larger slope means stomata close at higher RWC. Analysis showed that RWC50 and slope were strongly correlated (r^2^ = 0.94 for L and r^2^ = 0.96 for M plants). For that reason only data of slope are shown.

**Fig. 5.**
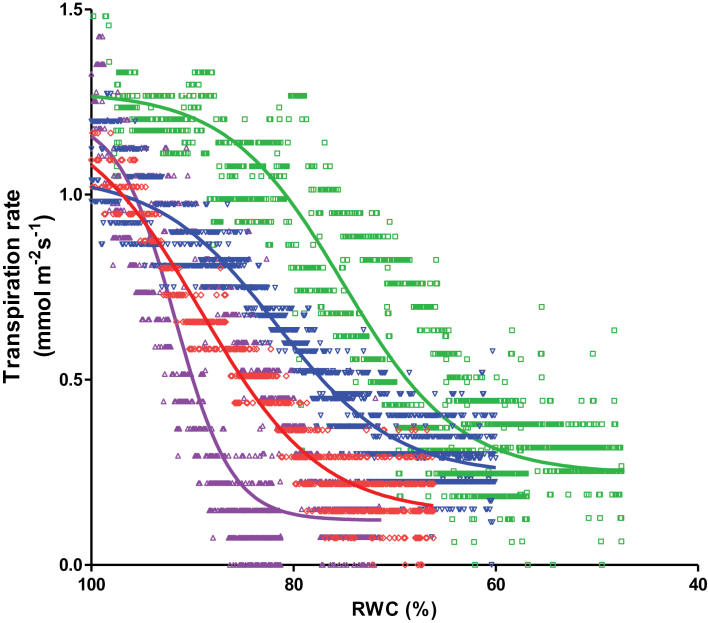
Fitted curves of the relationship between transpiration rate (E) and leaf relative water content (RWC) for Col-0 (red and blue lines) and Cvi-0 (purple and green lines) *Arabidopsis* accessions of leaves of plants that had been exposed for 4 d to moderate (red and purple symbols) or to low (blue and green symbols) VPD. The leaves were first saturated in degassed deionized water and after 1h measurements were conducted during desiccation at VPD of 1.40 kPa. The R square of goodness of fits was 0.9±0.1.

**Fig. 6.**
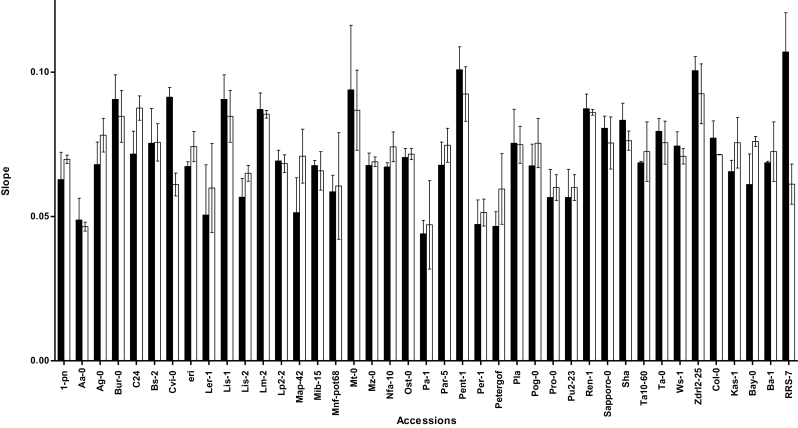
Slopes of the curves for relationship between transpiration rate (E) and leaf relative water content during 10 000 s desiccation of the leaves of plants that had been exposed for 4 d to moderate (1.17 kPa; filled bars) or to low (0.23 kPa; open bars) VPD. The leaves were first saturated in degassed deionized water and after 1h measurements were conducted during desiccation at VPD of 1.40 kPa.

The E×RWC for M- and L-exposed Col-0 and Cvi-0 are presented as examples (Fig. 5). Although L-exposed Col-0 exhibited higher E at certain RWC, no statistical difference were found for slope of the curves between M- and L-exposed Col-0 plants. Whereas, in the case of Cvi-0, slope of the E×RWC for L plants was significantly less compared with slope in M plants. Fig. 6 shows the slope of the E×RWC in all accessions when they had been previously exposed to M and L conditions. Most of the *Arabidopsis* accessions responded in the same way in both M- and L-exposed plants. In contrast to ABA, accessions were similarly distributed for their slope of E×RWC after exposure to M and L conditions (Supplementary Fig. S2). However, the slope for Cvi-0 and Rrs-7 was different between their M and L plants. Cvi-0 and Rrs-7 plants that been exposed to L condition showed slower rate for stomatal closure compared with M plants. This indicates that Cvi-0 and Rrs-7 plants lost more water in response to desiccation after prior exposure to L condition in comparison with M plants.

### Stomatal response to closing stimuli after prior exposure to different VPDs reveals natural variation among *Arabidopsis* accessions

**Fig. 7.**
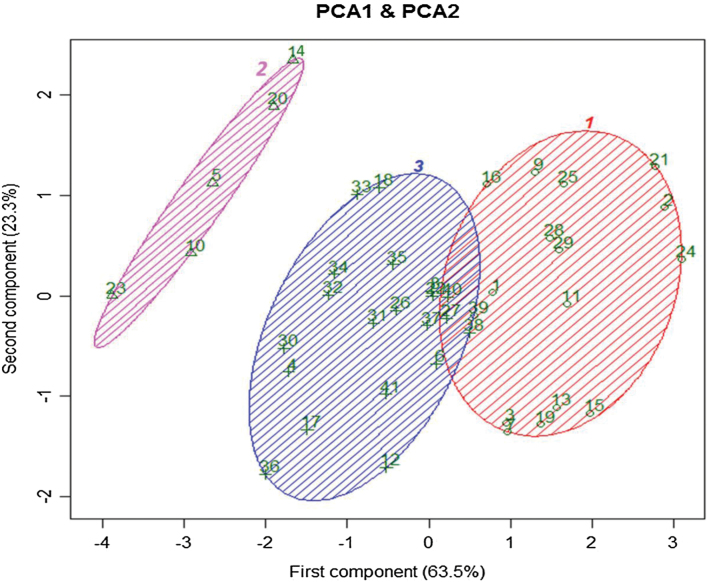
Principle component analysis (PCA) for 41 *Arabidopsis* accessions that had been exposed for 4 d to moderate VPD (1.17 kPa) or to low VPD (0.23 kPa). The numbers indicate the accessions according to the numbering in Table 1. The PSII efficiency (Φ_PSII_) under non-photorespiratory conditions at 200 µM ABA relative to Φ_PSII_ of the control (0 µM ABA), and the slope of the fitted sigmoidal relationship between transpiration rate and RWC of the leaves were used for the analysis. Component one and two explain 86.3% of the point variability.

**Fig. 8.**
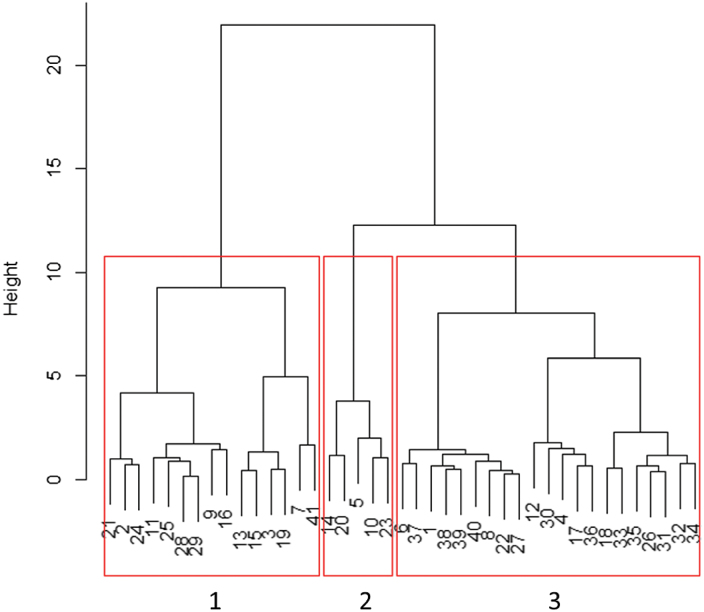
Dendrogram classification for 41 *Arabidopsis* accessions that had been exposed for 4 d to moderate VPD (1.17 kPa) or to low VPD (0.23 kPa). The PSII efficiency (Φ_PSII_) under non-photorespiratory conditions at 200 µM ABA relative to Φ_PSII_ of the control (0 µM ABA), and the slope of the fitted sigmoidal relationship between transpiration rate and RWC of the leaves were used for classification. The red boxes indicate accessions with three different type of responses to closing stimuli. The number at the bottom of the dendrograms correspond to the number of PCA grouping.

To group all tested accessions according to the effect of a prior exposure to different VPDs on their stomatal response to ABA and to desiccation, a global principle component analysis (PCA) was performed on the plants that had been exposed to M and L conditions. For the stomatal response to ABA, the relative effect of ABA on Φ_PSII_ was used and for the response of stomata to desiccation the slopes as given in Fig. 6 were used. The result showed that PCA1 and PCA2 explained 86.8% of the point variation between *Arabidopsis* accessions (Fig. 7). PCA1 accounted for 63.5% and PCA2 accounted for 23.3% of the observed variation. As the correlations between RWC50 and slope were more than 0.9 for both M and L plants, only the slope of the fitted curves was used for the PCA. The PCA showed that also adding g_s_ (stomatal conductance after exposure to M and L conditions) to the analysis did not increase the explained part of the point variation. The PCA showed three distinct groups for the stomatal responses to closing stimuli in all accessions when they had been previously exposed to M and L conditions (Fig. 7). Most of the accessions including Col-0 (accession number 37) belong to one group (number 3). Fig. 8 shows the classification of 41 accessions using cluster algorithms of the dataset. Group number 2 shows the accessions with extreme responses, Map-42, C24, Pent1, Lis1, and Ost-0, characterized as accessions with maximum response of stomata to closing stimuli, after prior exposure to moderate and low VPD. Moreover, two other big groups (numbers 1 and 3 in Fig. 7) can be categorized into two distinct clusters for their stomatal response to closing stimuli (Figs 7 and 8).

### VPD and desiccation considerably influenced foliar ABA level

**Fig. 9.**
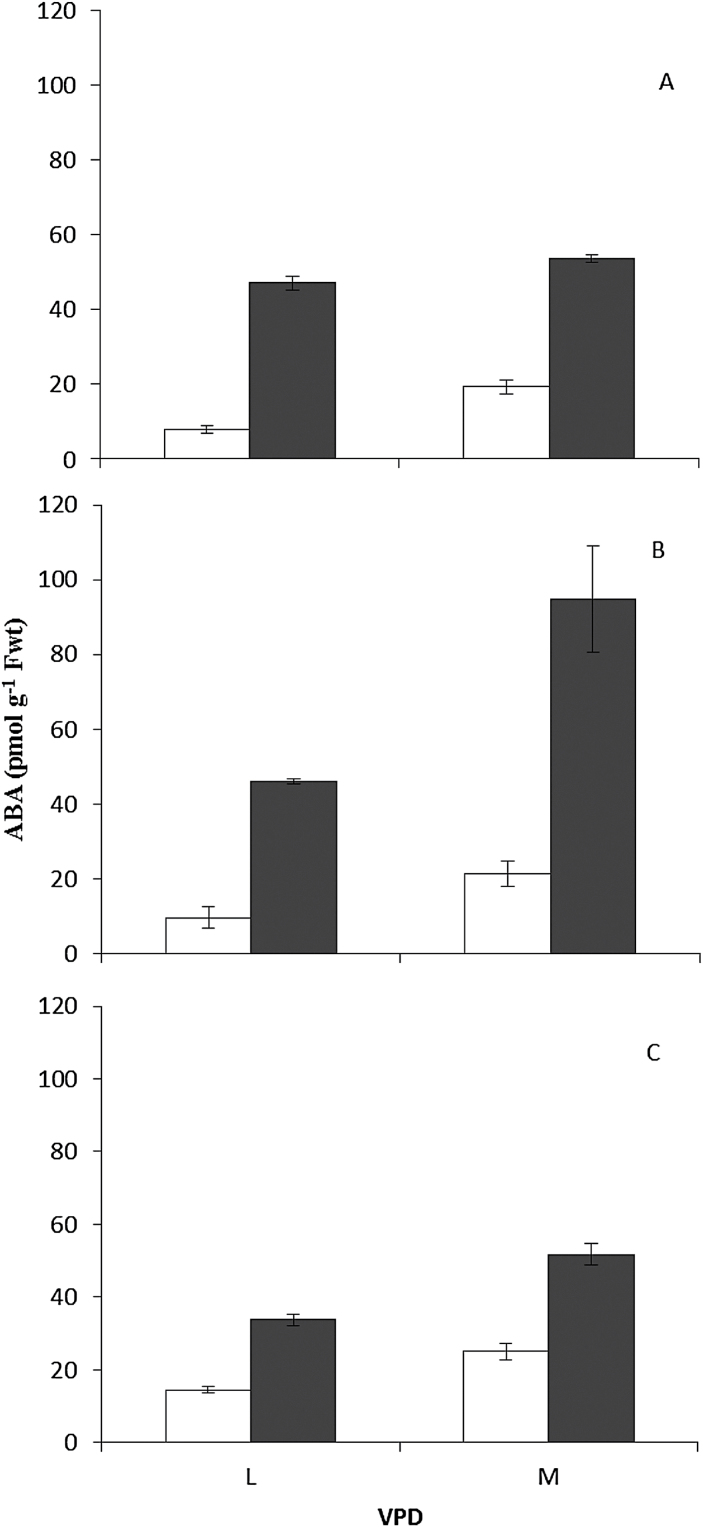
Concentration of ABA in Col-0 (A), Cvi-0 (B), and Map-42 (C) *Arabidopsis* accessions before (white bars) and after 45min desiccation (black bars). The plants had been exposed for 4 d to moderate VPD (M) (1.17 kPa) or to low VPD (L) (0.23 kPa) before ABA measurements and desiccation treatment. The desiccation was conducted at VPD of 1.40 kPa.

From the results obtained from screening of the stomatal response of *Arabidopsis* accessions to closing stimuli (after prior exposure to two different VPDs), two extreme accessions [Map-42 (group 2) and Cvi-0 (group1)] together with a ‘control’ accession [Col-0 (group3)] were used for measuring the bulk foliar ABA levels before and after desiccation. Before desiccation, lower ABA levels were found in the leaves of all three accessions as a result of exposure to L condition (Fig. 9; A, B, and C). After exposure to L condition the ABA level in the Map-42 (Fig. 9C) was 44% and 32% higher than the level in Col-0 and Cvi-0, respectively. Desiccation led to a sharp increase (*P*≤0.001) in the bulk foliar ABA level in all three accessions. In all three accessions, the level of ABA after desiccation was more in the plants that had been previously exposed to M condition, but there was a large difference in the after-effect of VPD on the increase in ABA owing to desiccation. In Col-0 the [ABA] in L plants was 88% of that of M plants after desiccation, whereas in L plants of Cvi-0 it was 49% of that of M plants. The highest bulk foliar ABA level following desiccation was found in the M-exposed Cvi-0 plants (Fig. 9B).

**Fig. 10.**
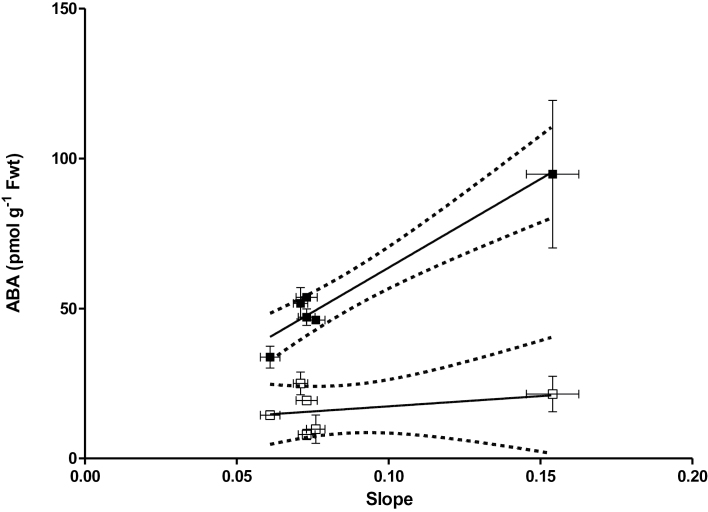
Relationship between desiccation response (slope of the E×RWC relationship) and the ABA-concentration before (open symbols) and after (closed symbols) 45min desiccation of the leaves in Col-0, Cvi-0, and Map-42 accessions. The dashed line is 95% confidence interval. R^2^ of the goodness of the fit is 0.94 for closed symbols and 0.12 for open symbols.

In these three accessions there was no significant correlation (*P*=0.49) between the desiccation response (slope of the E×RWC) and the foliar ABA level before desiccation (Fig. 10). However, slope of the E×RWC positively correlated (*P*=0.0012) with the amount of ABA produced owing to desiccation (Fig. 10).

**Fig. 11.**
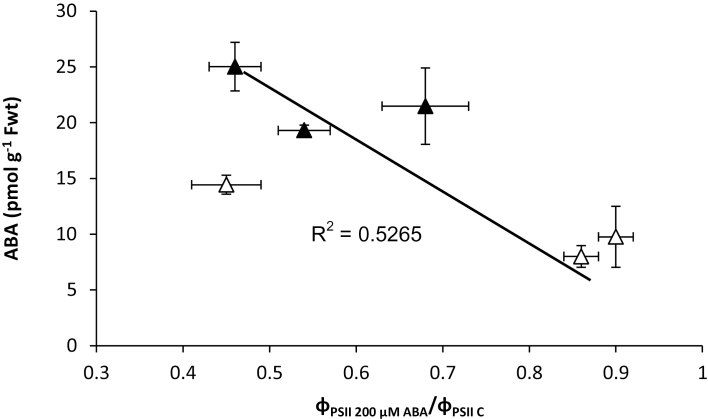
Relationship between PSII efficiency (Φ_PSII_) under non-photorespiratory conditions in response to 200 µM ABA relative to no ABA (Φ_PSII 200 ABA_/Φ_PSII C_) and foliar ABA level for plants that had been exposed for 4 d to moderate (1.17 kPa) (closed symbols) or to low VPD (0.23 kPa) (open symbols).

Of the 3 accessions tested, the response to ABA (Φ_PSII 200 ABA_/Φ_PSII C_) was inversely correlated to the foliar ABA level (before desiccation) of M and L plants (Fig 11). A high Φ_PSII 200 ABA_/Φ_PSII C_ indicates no closing of stomata.

## Discussion

### Fast screening procedure for ABA sensitivity of stomatal closing

To analyse the response of stomata to exogenous ABA, we developed an efficient and fast technique based on fluorescence of chlorophyll. In this technique, leaf discs were prepared from the leaves of plants that had been exposed to different VPDs and were floated in petri dishes (filled with stomata opening medium together with different concentrations of ABA). The PSII efficiency (Φ_PSII_) of the leaf discs was measured under non-photorespiratory conditions (low O_2_). In this situation, the only source for CO_2_ assimilation is the ambient CO_2_ which will be provided through stomata. Therefore, the closure of the stomata is the main reason for decreased Φ_PSII_ of the leaf discs. To test whether the decreased Φ_PSII_ is via stomatal closure, at the end an image was taken after 5min exposure to 50 000 ppm CO_2_ for recovering Φ_PSII_. The recovery of Φ_PSII_ by exposure to high CO_2_ confirmed that the decreased Φ_PSII_ is because of stomatal closure. In the imaging area of the system it was feasible to investigate 32 samples simultaneously. However, the relationship between CO_2_ assimilation and g_s_ is not always linear. Therefore, the developed method provides a fast and efficient way for investigating the qualitative response of the stomata to ABA.

### *Arabidopsis* showed remarkable natural genotypic variation for stomatal response to closing stimuli after prior exposure to different VPDs

Natural genetic variation between accessions is advantageous to study, because it allows an understanding of which processes within a trait are subjected to natural selection (Alonso-Blanco *et al.*, 2009; Trontin *et al.*, 2011). The response of stomata to environmental conditions is a complex trait involving a complex network of signalling pathways. Natural variations in plant sensitivity to ozone (Brosché *et al.*, 2010) and mild water stress (Bouchabke *et al.*, 2008) were reported among *Arabidopsis* accessions, which indirectly can be related to the stomata. In this study, we compared the stomatal response to closing stimuli after the plants had previously been exposed to moderate and low VPD conditions, to reveal natural variation among *Arabidopsis* accessions. We have demonstrated that there is remarkable natural variation among *Arabidopsis* accessions for adaptation (or disturbance) of the stomatal responses to closing stimuli after long-term exposure to low VPD. The studied accessions can be categorized in three different groups according to the adaptation of their stomatal response to ABA and desiccation by low VPD: sensitive to ABA and desiccation, sensitive to desiccation but not anymore to ABA, and non-sensitive to ABA and desiccation after low VPD-exposure.

### Outliers from screening of *Arabidopsis* accessions can be used to identify new molecular constituents involved in the stomatal response to closing stimuli

The results of our study revealed that there is a genotypic variation in the after-effect of long-term exposure to low VPD on the stomatal response among 41 distinct *Arabidopsis* accessions. The current screening revealed that Map-42 and C24 are accessions that maintained their response to ABA and desiccation, whereas Cvi-0 is an accession that lost its response to desiccation and ABA after prior exposure to low VPD. Most of the accessions, including Col-0, were recognized as responsive to desiccation but non-responsive to ABA after long-term exposure to L condition. To confirm that Map-42, C24, and Cvi-0 were outliers, the stomatal responses of these accessions to ABA and to desiccation were further analysed (2–4 times) as separate repetitions (data not shown). The plants were exposed to moderate and low VPD in other growth chambers as before to be sure that the effects were due to VPD and not to other differences between the growth chambers. If the stomatal response after long-term exposure to low VPD is controlled by an adaptive mechanism, the outliers with extreme responses can be used for building up promising RIL populations for identification of the involved QTLs in the malfunctioning stomata. QTL mapping for the stomatal response to environmental conditions are scarce. Screening 164 plants of a Col-0×Cvi-0 RIL population for ozone and water-loss phenotypes showed three QTLs for ozone and one QTL for water loss (Brosché *et al.*, 2010). The strongest QTL for ozone sensitivity was close to the same position as the QTL for water loss. Therefore, it is likely there is a correlation between stomatal functioning and plant injury response to ozone stress (Brosché *et al.*, 2010). In agreement with our study, natural genetic variation was found between 24 accessions of *Arabidopsis* under a water-deficit condition (Bouchabke *et al.*, 2008). The mentioned studies are the only ones that showed a variation in response of the plants to environmental conditions that were indirectly related to variation in stomatal functioning. Natural variation in stomatal density and stomatal index has been found among 62 wild *Arabidopsis* accessions (Delgado *et al.*, 2011). However, stomatal morphological alterations owing to long-term exposure to L condition is not the main reason for stomatal malfunctioning after exposure to low VPD (Aliniaeifard *et al.*, 2014). To the best of our knowledge, the current study is the only one which focuses on the natural variation in stomatal response of *Arabidopsis* accessions when they have been exposed for long-term to low VPD conditions. RILs from different *Arabidopsis* parents (such as RIL populations for Col-0, Ler-1, Cvi-0, C24, and Te-0) have been used for QTL mapping for traits such as flowering time, seed dormancy, and resistance to disease which participate in plant response and adaptation to different environmental conditions (Shindo *et al.*, 2007; Brosché *et al.*, 2010). If the variation in stomatal response is at least partially because of selection pressure, it would be reasonable to find some correlation between the stomatal sensitivity to closing stimuli and the environment where the accessions originally come from. Significant differences were found for the relationship between g_s_ of moderate (*P*=0.006) and low (*P*=0.004) VPD-exposed plants and precipitation (Supplementary Fig. S4), However, no correlations were found between geographical parameters and stomatal sensitivity to ABA and desiccation for *Arabidopsis* accessions. Therefore, most probably genetic drift is the reason for the observed variation between accessions. The recognized natural variation in the current study can be useful for finding genes and signalling pathways involved in the malfunctioning of stomata due to low VPD.

### Low VPD condition reduced the stomatal response to ABA, but did not highly affect stomatal response to desiccation

The disturbed ABA signalling pathway due to long-term exposure to low VPD was reviewed by Aliniaeifard and van Meeteren (2013) in more detail. The results of the current study showed that most of the *Arabidopsis* accessions were not capable of full stomatal closure in response to different ABA concentrations after exposure to L (Supplementary Table S2). As a result of long-term exposure to low VPD, habituation occurs which renders the stomata insensitive to ABA (Aliniaeifard and van Meeteren, 2013).

In response to desiccation most of the *Arabidopsis* accessions showed stomatal closure after exposure to both moderate and low VPDs. However in this study, Cvi-0 was recognized as an accession with malfunctioning stomata in response to desiccation after prior exposure to low VPD. Compared with other *Arabidopsis* accessions Cvi-0 had the highest stomatal conductance after exposure to different VPDs (Fig. 2). It has been shown that high stomatal conductance in Cvi-0 caused a high rate of ozone uptake by the leaf, resulting in more sensitivity of this accession to ozone (Brosché *et al.*, 2010). Moreover, long-term exposure to ozone reduced the sensitivity of the stomata in response to different closing stimuli (Paoletti, 2005; Mills *et al.*, 2009; Wilkinson and Davies, 2009; Aliniaeifard and van Meeteren, 2013), resulting in more damage by ozone in the long term. Bouchabke *et al.* (2008) showed that compared with 23 other *Arabidopsis* accessions, Cvi-0 had the highest leaf water loss in well-watered and water-deficit conditions. In our study, L-exposed Cvi-0 lost more water compared with M-exposed Cvi-0. Similar to the current study with prior exposure to different VPDs, a difference between well-watered and water-deficit grown Cvi-0 plants was found in water loss after two hours desiccation (Bouchabke *et al.*, 2008). QTL mapping in a core Col-0×Cvi-0 RIL population identified one QTL for high water loss trait (Bouchabke *et al.*, 2008).

E, apart from g_s_, also depends on boundary layer conductance (g_b_) and cuticular conductance (g_c_). As the irradiance that was used for the desiccation response of the accessions was much lower than the irradiance in the growth chambers, we can expect a relatively low g_s_ during the desiccation experiment. As a result, the role of g_b_ and g_c_ during the desiccation experiment can be relatively large. The duration of exposure to low VPD (4 d) was not long enough to influence the cuticular characteristics; however, there is the possibility of involvement of g_b_ and g_c_ differences among accessions on the slope of the RWC×E curves.

Why are most of the *Arabidopsis* accessions still responsive to desiccation after exposure to low VPD although they lost their responsiveness to ABA? Analysing the stomatal response of four different rose cultivars, Fanourakis *et al.* (2013) showed that in one of the cultivars stomatal response to exogenous ABA was considerably influenced by growth at low VPD, whereas its response to desiccation was only minimally affected. In a study using full-grown leaves of bean plants, Aliniaeifard *et al.* (2014) found that as a result of exposure to low VPD, stomatal responsiveness to ABA was decreased before a diminished response to desiccation occurred. They concluded that the stomatal responses to desiccation and to ABA were not affected in the same way by exposure to low VPD. They suggested that signals induced by desiccation were capable of increasing ABA levels in the guard cells, but ABA feeding to the petiole was not or that desiccation controls stomata closure (also) via a non-ABA-controlled pathway (Munns and King, 1988; Aliniaeifard *et al.*, 2014). Exposure to different VPDs affected g_s_ as the desiccation response (slope of RWC×E) of the ecotypes, but these changes (g_sL_/g_sM_ and slope_L_/slope_M_) were not correlated (*P*=0.338) to each other (Supplementary Fig. S3A). This may also highlight the involvement of g_b_ and g_c_ differences among accessions on the E during desiccation experiments. Nevertheless, although the correlation was not strong, the effect of VPD on the stomatal response to ABA significantly correlated (*P*=0.005) positively with the effect of VPD on g_s_ (Supplementary Fig. S3B). This strengthens the concept that desiccation controls stomata closure (also) via a non-ABA controlled pathway. It has been shown that ABA is not the only signal causing stomatal closure under water shortage conditions (Holbrook *et al.*, 2002; Munns and King, 1988).

### Stomatal conductance is an important indicator of stomatal response to ABA

In general, prior exposure to low VPD led to higher stomatal conductance and less stomatal response to ABA. Similarly, increased stomatal conductance and decreased stomatal responsiveness to ABA owing to long-term exposure to low VPD has been reported in *Vicia faba* (Aliniaeifard *et al.*, 2014), *Tradescantia virginiana* (Rezaei Nejad and van Meeteren, 2005, 2007) and *Rosa hybrida* (Fanourakis *et al.*, 2011; Fanourakis *et al.*, 2013).

The involvement of ABA in the stomatal response to water stress is extensively studied. It has been well documented that drought induced ABA production results in stomatal closure (Larque-Saavedra and Wain, 1974; Luan, 2002; Giday *et al.*, 2013). Accordingly, in the current study a positive correlation (R^2^=0.94) was found between foliar ABA level after desiccation and transpiration rate (slope of E×RWC). In *Vicia faba* and *Tradescantia* long-term exposure to low VPD decreased the ABA level and thereafter stomata are no longer responsive to closing stimuli (Rezaei Nejad and van Meeteren, 2008; Aliniaeifard *et al.*, 2014). It was concluded that low foliar ABA level for long time could be the main reason for malfunctioning of the stomata in response to closing stimuli (Rezaei Nejad and van Meeteren, 2007; Aliniaeifard and van Meeteren, 2013). After exposure to low VPD, foliar ABA level decreased via ABA 8’-hydroxylases (Kushiro *et al.*, 2004; Okamoto *et al.*, 2009). It was suggested that as a result of long-term low ABA level, the ABA receptors are unable to block ABA-negative regulators inside the guard cells, which consequently leads to stomatal insensitiveness to ABA (Rezaei Nejad and van Meeteren, 2007; Aliniaeifard and van Meeteren, 2013). Overcoming the low ABA level due to exposure to low VPD via daily application of ABA during leaf development in rose (*Rosa hybrida*) (Fanourakis *et al.*, 2011) and *Tradescantia* (Rezaei Nejad and van Meeteren, 2007) or during 4 d exposure to low VPD in *Vicia faba* (Aliniaeifard *et al.*, 2014) maintained functional stomata that are responsive to closing stimuli (e.g. ABA). In the three accessions tested, as representatives of the three clusters of the PCA, a positive correlation was found between foliar ABA level and stomatal closure response to ABA after exposure to different VPDs (Fig. 11). Cvi-0 showed the largest decrease in foliar ABA level after exposure to low VPD (Fig. 9) and lost its response to ABA, whereas MAP-42 showed the smallest decrease of ABA and kept its response to ABA after low VPD-exposure.

In conclusion, we have shown that there is natural variation in the effect of long-term exposure to low VPD on the sensitivity to closing stimuli among 41 accessions of *Arabidopsis thaliana*. This variation can be exploited to identify genes involved in the signalling pathways in malfunctioning stomata, owing to long-term exposure to low VPD. Accessions can be categorized in three groups according to their stomatal response to closing stimuli after prior exposure to low VPD. Stomata of most of the *Arabidopsis* accessions were not fully responsive to ABA when the plants had been exposed to low VPD, but most of them were responsive to desiccation after exposure to low VPD. Stomatal response to ABA, but not to desiccation, was related to the stomatal conductance after exposure to low VPD.

## Supplementary data

Supplementary data are available at JXB online.

Table S1. Composition of nutrient solution.

Table S2. The effect of different ABA concentrations on PSII efficiency (Φ_PSII_) under non-photorespiratory conditions for 41 *Arabidopsis* accessions that had been exposed to different VPDs.

Figure S1. Transpiration rate (E) for Col-0 and Cvi-0 *Arabidopsis* accessions during desiccation of leaves of plants that had been exposed to different VPDs.

Figure S2. Distribution of 41 *Arabidopsis* accessions that had been exposed to different VPDs according to the slope of E×RWC during desiccation of the leaves.

Figure S3. Relation between the effect of prior VPD-exposure on stomatal conductance (g_s_) and closing stimuli in 41 *Arabidopsis* accessions.

Figure S4. Relation between the effect of a 4-day VPD-exposure on stomatal conductance (g_s_) and average seasonal precipitation for 41 *Arabidopsis* accessions. Plants had been exposed to moderate VPD (1.17 kPa; open symbols) or to low (0.23 kPa; filled symbols) VPD. Measurements of g_s_ were conducted at a VPD of 1.40 kPa.

Supplementary Data
