# Supplementary material for: Natural variation in stomatal response to closing stimuli among Arabidopsis thaliana accessions after exposure to low VPD as a tool to recognize the mechanism of disturbed stomatal functioning
Source: J Exp Bot. 2014 Sep 9;65(22):6529–42. doi: 10.1093/jxb/eru370 (PMC4246184; doi:10.1093/jxb/eru370)
Supplement: Supplementary Data [file supp_65_22_6529__index.html]

Natural variation in stomatal response to closing stimuli among Arabidopsis thaliana accessions after exposure to low VPD as a tool to recognise the mechanism of disturbed stomatal functioning — Natural variation in stomatal response to closing stimuli among Arabidopsis thaliana accessions after exposure to low VPD as a tool to recognize the mechanism of disturbed stomatal functioning — Supplementary Data 

# Natural variation in stomatal response to closing stimuli among *Arabidopsis thaliana* accessions after exposure to low VPD as a tool to recognize the mechanism of disturbed stomatal functioning

## Supplementary Data

Data files

**Files in this Data Supplement:**

- Supplementary Data - Supplementary Data
