# Supplementary material for: Natural variation in stomatal response to closing stimuli among Arabidopsis thaliana accessions after exposure to low VPD as a tool to recognize the mechanism of disturbed stomatal functioning
Source: J Exp Bot. 2014 Sep 9;65(22):6529–42. doi: 10.1093/jxb/eru370 (PMC4246184; doi:10.1093/jxb/eru370)
Supplement: Supplementary Data [file supp_eru370_jexbot130740_file001.pdf]

Title: Natural variation in stomatal response to closing stimuli among *Arabidopsis thaliana* accessions after exposure to low VPD as a tool to recognise the mechanism of disturbed stomatal functioning

Authors' names: Sasan Aliniaeifard and Uulke van Meeteren

Table S1. Composition of nutrient solution

Macro elements		Micro elements	
**NH_4 (mmol/L)**	1.7	**Na (mmol/L)**	0.4
**K (mmol/L)**	4.5	**Cl (mmol/L)**	0.2
**Ca (mmol/L)**	2.3	**Si (mmol/L)**	0.23
**Mg (mmol/L)**	1.5	**Fe (μmol/L)**	21
**NO_3 (mmol/L)**	4.4	**Mn (μmol/L)**	3.4
**S (mmol/L)**	3.5	**Zn (μmol/L)**	4.7
**P (mmol/L)**	1.12	**B (μmol/L)**	14
**HCO_3(mmol/L)**	0.6	**Cu (μmol/L)**	6.9
		**Mo (μmol/L)**	<0.1
**EC (Ms/cm)= 1.4, pH= 7.0**			

Table S2. The effect of different ABA concentrations (50, 100, 200 µM) on PSII efficiency (Φ_{PSII}) under non-photorespiratory conditions for 41 *Arabidopsis* accessions which have been exposed for 4 days to moderate (1.17 kPa; M) or to low (0.23 kPa; M) VPD. Φ_{PSII} is expressed as relative effect of ABA to the control treatment (without ABA). Leaf discs (0.5 cm diameter) were put with the abaxial surface down in petri dishes with stomata-opening medium with different ABA concentrations and Φ_{PSII} was recorded 3 hr after application of ABA. Numbers are mean values of 8 leaf disks ±SEM.

Accession	PCA number	VPD	$\varphi_{PSII\ 50\ \mu M\ ABA}/\varphi_{PSII\ C}$	$\varphi_{PSII\ 100\ \mu M\ ABA}/\varphi_{PSII\ C}$	$\varphi_{PSII\ 200\ \mu M\ ABA}/\varphi_{PSII\ C}$
**1-pn**	1	M	0.73±0.04	0.67±0.03	0.58±0.05
		L	0.91±0.01	0.93±0.02	0.89±0.01
**Aa-0**	2	M	0.80±0.02	0.74±0.01	0.67±0.03
		L	0.91±0.01	0.91±0.03	0.90±0.03
**Ag-0**	3	M	0.83±0.02	0.76±0.02	0.70±0.03
		L	0.92±0.02	0.90±0.02	0.89±0.03
**Bur-0**	4	M	0.66±0.03	0.66±0.04	0.56±0.05
		L	0.82±0.03	0.80±0.02	0.68±0.04
**C24**	5	M	0.63±0.08	0.54±0.04	0.46±0.03
		L	0.64±0.07	0.56±0.07	0.49±0.05
**Bs-2**	6	M	0.71±0.05	0.68±0.04	0.60±0.05
		L	0.88±0.03	0.86±0.03	0.85±0.03
**Cvi-0**	7	M	0.87±0.03	0.77±0.03	0.68±0.05
		L	0.92±0.02	0.92±0.02	0.90±0.02
**Eri-1**	8	M	0.75±0.03	0.67±0.04	0.55±0.05
		L	0.91±0.01	0.86±0.04	0.83±0.03
**Ler-1**	9	M	0.85±0.02	0.62±0.03	0.51±0.04
		L	0.93±0.01	0.92±0.02	0.91±0.01
**Lis-1**	10	M	0.51±0.03	0.49±0.04	0.43±0.04
		L	0.63±0.02	0.64±0.03	0.58±0.04
**Lis-2**	11	M	0.86±0.03	0.75±0.04	0.66±0.04
		L	0.92±0.03	0.89±0.03	0.89±0.03
**Lm-2**	12	M	0.81±0.05	0.67±0.03	0.62±0.04
		L	0.94±0.02	0.91±0.05	0.86±0.03
**Lp2-2**	13	M	0.86±0.02	0.85±0.03	0.72±0.07
		L	0.95±0.02	0.95±0.03	0.91±0.02
**Map-42**	14	M	0.64±0.03	0.54±0.04	0.45±0.03
		L	0.65±0.04	0.56±0.03	0.46±0.04
**Mib-15**	15	M	0.83±0.04	0.81±0.03	0.73±0.05
		L	0.98±0.02	0.94±0.03	0.96±0.02
**Mnf-pot68**	16	M	0.66±0.07	0.64±0.05	0.57±0.04
		L	0.84±0.05	0.84±0.03	0.75±0.05

**Mt-0**	17	M	0.71±0.03	0.58±0.05	0.55±0.06
		L	0.93±0.02	0.89±0.02	0.80±0.05
**Mz-0**	18	M	0.59±0.04	0.58±0.04	0.49±0.04
		L	0.76±0.06	0.79±0.03	0.71±0.06
**Nfa-10**	19	M	0.71±0.04	0.74±0.02	0.72±0.05
		L	0.92±0.02	0.92±0.02	0.91±0.02
**Ost-0**	20	M	0.49±0.05	0.44±0.01	0.42±0.03
		L	0.69±0.04	0.58±0.04	0.55±0.03
**Pa-1**	21	M	0.79±0.05	0.68±0.04	0.62±0.07
		L	0.93±0.02	0.95±0.01	0.93±0.01
**Par-5**	22	M	0.66±0.04	0.70±0.05	0.53±0.05
		L	0.88±0.03	0.89±0.02	0.87±0.02
**Pent-1**	23	M	0.48±0.02	0.47±0.03	0.40±0.03
		L	0.64±0.08	0.66±0.04	0.54±0.04
**Per-1**	24	M	0.80±0.05	0.76±0.04	0.70±0.06
		L	0.97±0.02	0.99±0.03	0.94±0.02
**Petergof**	25	M	0.77±0.04	0.66±0.05	0.59±0.04
		L	0.90±0.02	0.88±0.03	0.84±0.02
**Pla**	26	M	0.72±0.02	0.63±0.05	0.56±0.04
		L	0.90±0.03	0.80±0.04	0.77±0.03
**Pog-0**	27	M	0.73±0.03	0.62±0.04	0.55±0.04
		L	0.91±0.03	0.91±0.02	0.88±0.03
**Pro-0**	28	M	0.80±0.03	0.69±0.05	0.60±0.05
		L	0.90±0.03	0.91±0.03	0.86±0.02
**Pu2-23**	29	M	0.78±0.05	0.72±0.06	0.61±0.06
		L	0.86±0.03	0.88±0.01	0.87±0.01
**Ren-1**	30	M	0.64±0.07	0.64±0.03	0.46±0.03
		L	0.89±0.02	0.84±0.05	0.79±0.04
**Sapporo-0**	31	M	0.78±0.03	0.73±0.02	0.56±0.03
		L	0.88±0.02	0.81±0.03	0.75±0.03
**Shahdara**	32	M	0.73±0.02	0.64±0.04	0.50±0.03
		L	0.84±0.02	0.80±0.03	0.73±0.03
**Ta10-60**	33	M	0.61±0.07	0.54±0.04	0.45±0.03
		L	0.81±0.03	0.779±0.04	0.74±0.06
**Ta-0**	34	M	0.69±0.06	0.63±0.02	0.54±0.03
		L	0.77±0.04	0.71±0.05	0.65±0.06
**Ws-0**	35	M	0.74±0.04	0.62±0.03	0.53±0.04
		L	0.89±0.03	0.81±0.06	0.75±0.03
**Zdrl2-25**	36	M	0.77±0.05	0.63±0.03	0.55±0.04
		L	0.88±0.03	0.82±0.04	0.78±0.03
**Col-0**	37	M	0.84±0.02	0.65±0.03	0.54±0.03
		L	0.92±0.02	0.90±0.02	0.86±0.02
**Kas-1**	38	M	0.81±0.04	0.70±0.07	0.57±0.06
		L	0.96±0.03	0.93±0.04	0.90±0.05
**Bay-0**	39	M	0.84±0.03	0.70±0.06	0.59±0.03
		L	0.91±0.04	0.89±0.04	0.86±0.03

**Ba-1**	40	M	0.88±0.02	0.69±0.05	0.51±0.03
		L	0.95±0.02	0.93±0.02	0.92±0.03
**RRS-7**	41	M	0.75±0.03	0.73±0.05	0.59±0.03
		L	0.92±0.01	0.90±0.03	0.78±0.02

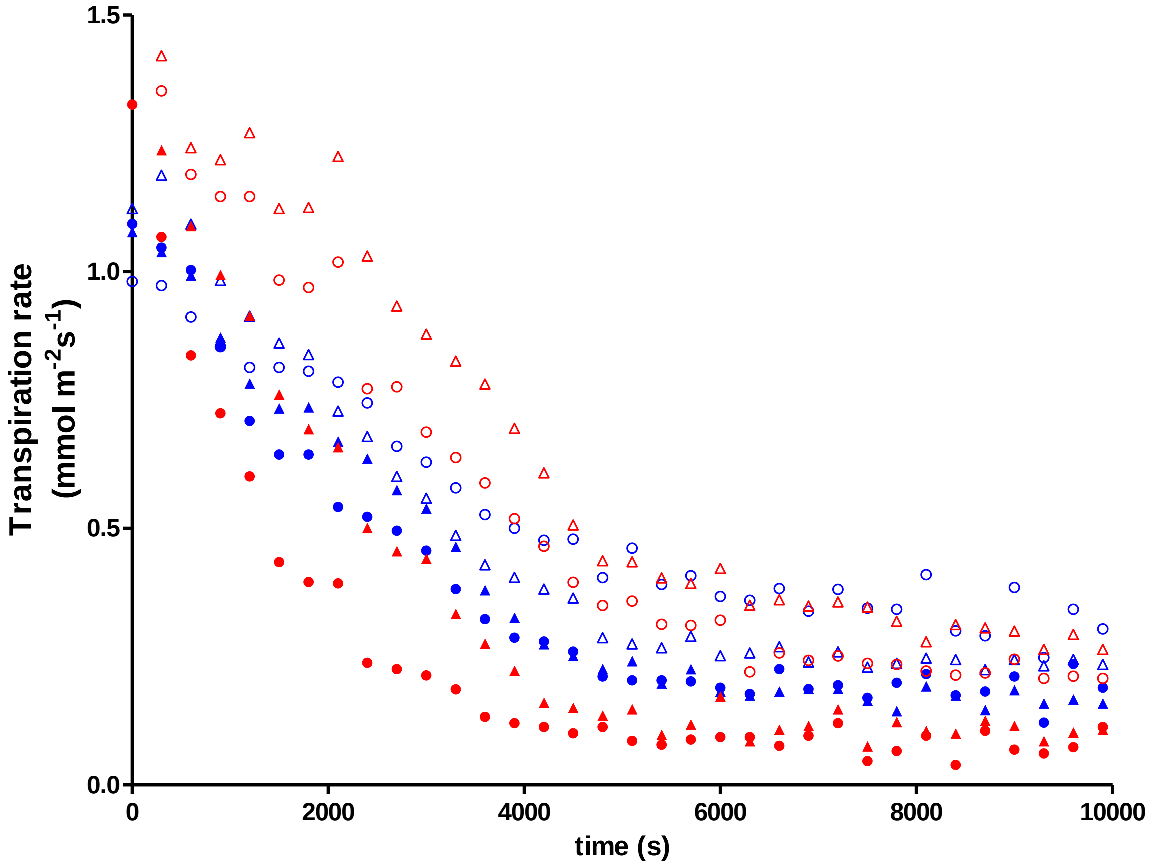

Figure S1. Transpiration rate (E) for Col-0 (blue symbols) and Cvi-0 (red symbols) *Arabidopsis* accessions during 10000 s desiccation of leaves of plants that have been exposed for 4 days to moderate (1.17 kPa; filled symbols) or to low (0.23 kPa; open symbols) VPD. The leaves were first saturated in degassed deionized water and after 1 hr measurements were conducted at VPD of 1.40 kPa. Circle and triangle symbols showing different repetitions.

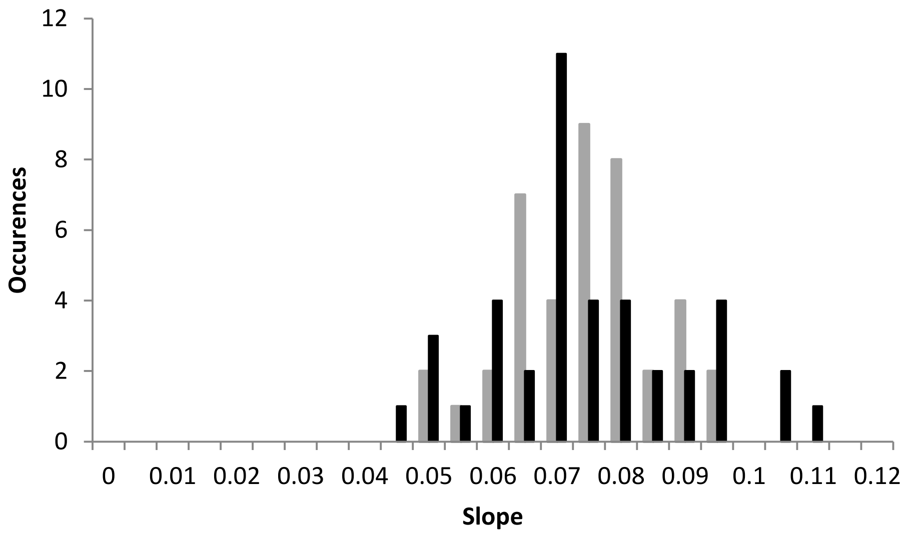

Figure S2. Distribution of 41 *Arabidopsis* accessions that have been exposed for 4 days to moderate (1.17 kPa; black bars) or to low (0.23 kPa; grey bars) VPD according to Slope of E×RWC during 10000 s desiccation of the leaves. The leaves were first saturated in degassed deionized water and after 1 hr measurements were conducted at VPD of 1.40 kPa.

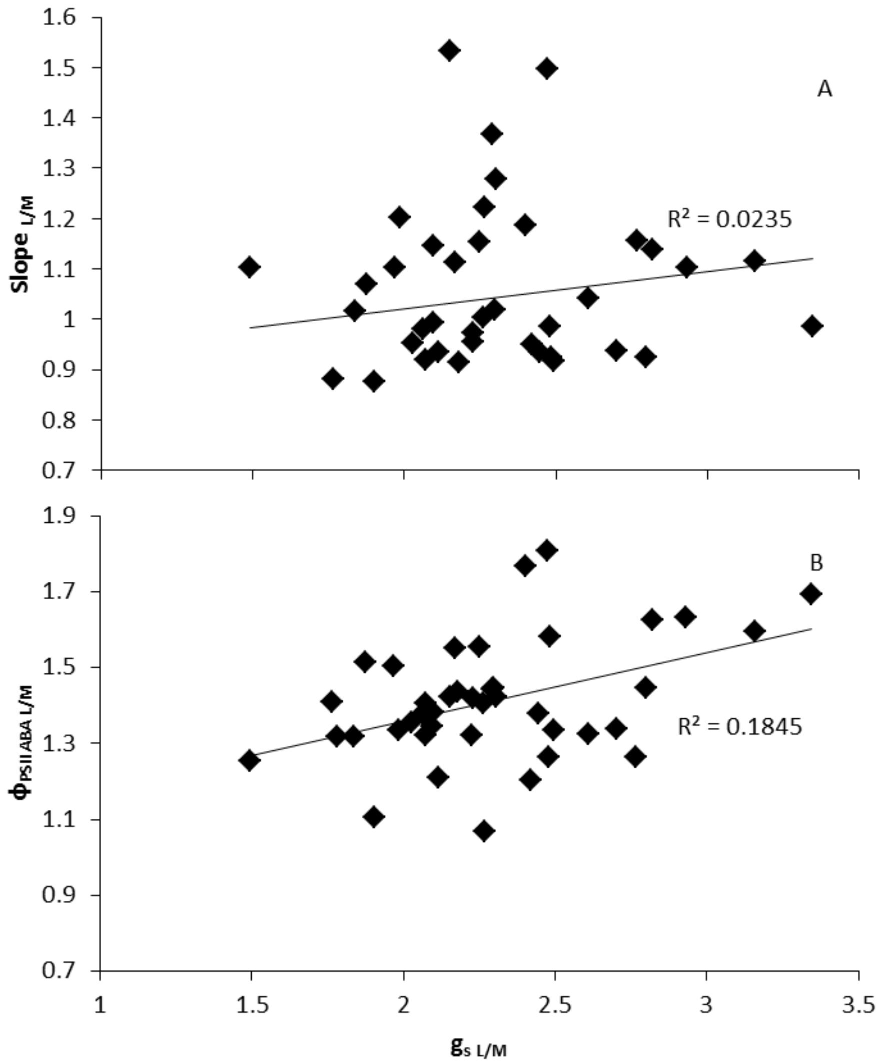

Figure S3. Relation between the effect of prior VPD-exposure on stomatal conductance (g_s) and desiccation response (a) or ABA response (b) of 41 *Arabidopsis* accessions. Plants had been exposed for 4 days to low VPD (0.23 kPa) or to moderate VPD (1.17 kPa). The effect of prior VPD on stomatal conductance was expressed as ratio between g_s at L/g_s at M ($g_{s\ L/M}$), on desiccation response as the ratio of the Slopes of RWC×E at L and M (Slope $_{L/M}$), and on ABA response as the ratio of the relative effects of 200 µM ABA to Φ_{PSII} ($\Phi_{PSII\ 200\ ABA}$ / $\Phi_{PSII\ C}$) at low and moderate VPD ($\Phi_{PSII\ ABA\ L/M}$). Measurements of g_s were conducted at a VPD of 1.40 kPa.

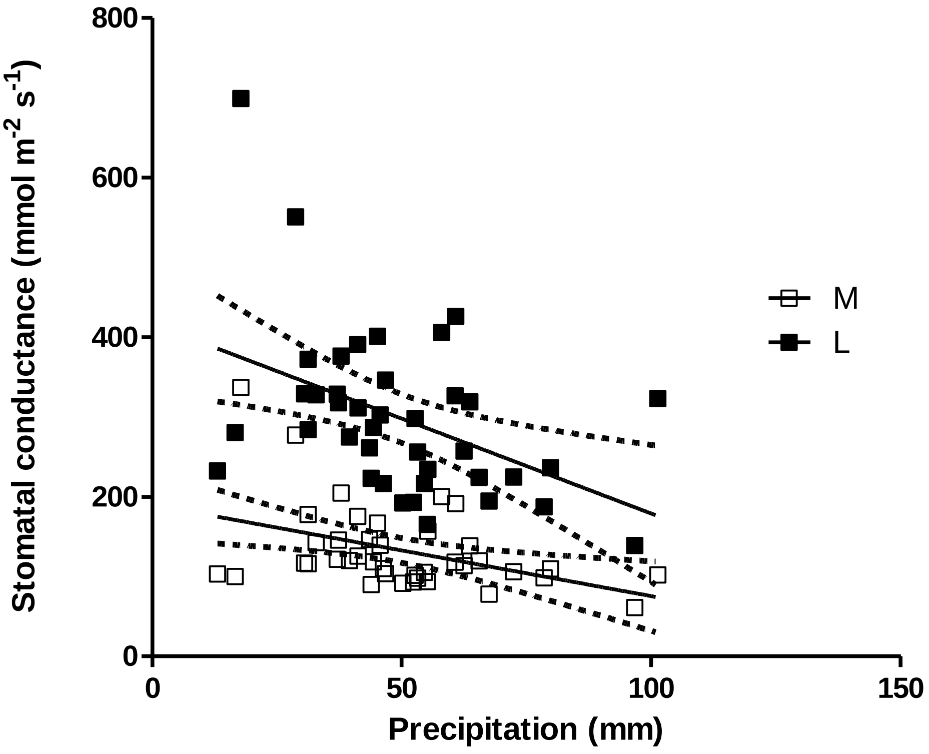

Figure S4. Relation between the effect of 4-day VPD-exposure on stomatal conductance (g_s) and average seasonal precipitation for 41 *Arabidopsis* accessions. Plants had been exposed to moderate VPD (1.17 kPa; open symbols) or to low (0.23 kPa; filled symbols) VPD. Measurements of g_s were conducted at a VPD of 1.40 kPa.
